# Does Aromatase Inhibitors Cause Sjogren’s Syndrome and Polyneuropathy?

**DOI:** 10.14740/wjon695w

**Published:** 2014-08-25

**Authors:** Nazife Sule Yasar Bilge, Cengiz Korkmaz

**Affiliations:** a Department of Internal Medicine, Division of Rheumatology, Eskisehir Yunus Emre State Hospital, Eskisehir, Turkey; b Department of Internal Medicine, Division of Rheumatology, Eskisehir Osmangazi University, Eskisehir, Turkey

**Keywords:** Sjogren’s syndrome, Breast cancer, Aromatase inhibitors

## Abstract

Aromatase inhibitors (AIs) are the indispensible part of hormone-responsive breast cancer treatment. A potential relation between autoimmunity and AIs has been described before. Herein, we report a case of Sjogren’s syndrome (SjS) and polyneuropathy which developed during treatment with anastrozole. A 70 years old female patient having a history of breast cancer was referred to our clinic with numbness in both legs for 1 year. She was receiving anastrazole since 2006. She was having sicca symptoms for 3 years. After laboratory evaluation, salivary gland biopsy and electroneuromyography, the patient was evaluated as SjS and polyneuropathy. Intravenous immunoglobulin treatment (400 mg/kg/day, 5 days, 6 months) was initiated. A potential pathogenic linkage between AI therapy and autoimmunity is mentioned. Only few cases of rheumatoid arthritis and SjS related with AIs have been reported. But, our case is the first in the literature having definite SjS and neuropathy in this setting.

## Introduction

Aromatase inhibitors (AIs) are the cornerstones of hormone-responsive breast cancer treatment. A pathogenic linkage between autoimmunity and AIs has been described before [1]. Here, we report a case of Sjogren’s syndrome (SjS) and polyneuropathy which developed during treatment with anastrozole, a third generation AI.

## Case Report

A 70 years old female patient was referred to our clinic with numbness in both legs for 1 year. She had a history of breast cancer (invasive ductal carcinom, T2N0Mx) for 6 years and right modified radical mastectomy was performed. She was receiving anastrazole since 2006 after six cycles of chemotherapy (fluorouracil, adriamycin, and cyclophosphamid). She was having sicca symptoms for 3 years. On physical examination, she had dry mouth and superficial sensorial loss in both legs. Her erythrocyte sedimentation rate was 47 mm/h (0 - 20) and C-reactive protein level was 8.87 mg/dL (0 - 8), respectively. Her rheumatoid factor and anti-nuclear antibody (ANA) tests were positive whereas SS-A and SS-B antibodies were negative. Schirmer test was 2 mm in right eye and 3 mm in left eye which was concordant with a positive test. Minor salivary gland biopsy was performed and the results were reported as compatible with SjS. Electroneuromyography showed axonal polyneuropathy. The patient was evaluated as SjS and polyneuropathy. Intravenous immunoglobulin treatment (400 mg/kg/day, 5 days, 6 months) was initiated. Until now she received three cycles and her symptoms improved moderately.

## Discussion

The third generation AIs, anastrozole, letrozole and exemestane, have become an important part of both early and advanced hormone-responsive breast cancer treatment in postmenopausal women [2]. They decrease levels of circulating estrogen in postmenopausal women by blocking the action of the aromatase enzyme, which converts androgens to estrogens. The main adverse effects of AIs are reduction of bone mineral density resulting in osteopenia, osteoporosis and fractures, joint symptoms, sexual dysfunction, and reactivation of ovarian function in premenopausal woman [1, 2]. Hormone receptor positivity, obesity, prior chemotherapy and treatment with anastrozole are associated with a higher risk of joint symptoms which may result with interruption of treatment [3]. Musculoskeletal symptoms related to AI therapy include arthralgia, morning stiffness, tenosynovitis, trigger finger and carpal tunnel syndrome. Estrogen deprivation has been accused as the cause of AI-related joint symptoms. A potential pathogenic linkage between AI therapy and autoimmunity is mentioned. Only few cases of rheumatoid arthritis (RA) and SjS related with AIs have been reported [4, 5]. Most of them were cases of incomplete SjS and none of them had neuropathy. In a study by Laroche et al, 24 patients presented with pain while receiving AI therapy were evaluated and 10 patients had sicca symptoms, nine patients had ANA positivity and one had anti-SSA and anti-SSB antibodies positivity. Of the 24 patients, 19 had arthralgia related to AIs but only one had definite RA and one other had definite SjS [5]. An association between estrogen deficiency and increased proinflammatory cytokine secretion is thought to be responsible. Studies about sex hormones and SjS have conflicting results about their relations, making it an intriguing topic [5].

Our case is the first in the literature having definite SjS and neuropathy in this setting. Some scenarios can be suggested for explaining the occurrence of SjS in this patient. SjS and neuropathy may be an adverse effect of chemotherapy which, we believe, is a remote possibility because 6-year interval between chemotherapy administration and occurrence of SjS is a very long period. Another explanation is that autoimmunity as a part of paraneoplastic process due to recurrence of malignancy could have developed. However, the patient was evaluated for this possibility, but no evidence for a relapse was found by oncology department. It is well known that cryoglobulinemia-related vasculitis may cause neuropathy in the course of SjS. The main manifestation of cryoglobulinemia-related vasculitis is palpable purpurae. In this patient, we did not observe any skin lesions that may suggest cryoglobulinemia-related vasculitis. Therefore we did not evaluate the presence of cryoglobulinemia.

Another mechanism is that AI could have caused SjS and neuropathy or triggered the onset of subclinical SjS in this patient. SjS may have mostly subclinical course, and the diagnosis of SjS is delayed because of this subclinical course. Although it is difficult to remember past medical complaints by the patient, our patient had no SjS-related symptoms prior to the development of breast cancer or when breast cancer was diagnosed. Therefore, it can be speculated that there may be a causal relationship between AI use and SjS and neuropathy. All her symptoms started under the treatment with AI. Presumably, further cases will clarify whether there is a causal relationship between AI use and autoimmune disorders such as SjS and shed light on the pathogenesis of AI-related autoimmune disorders.
